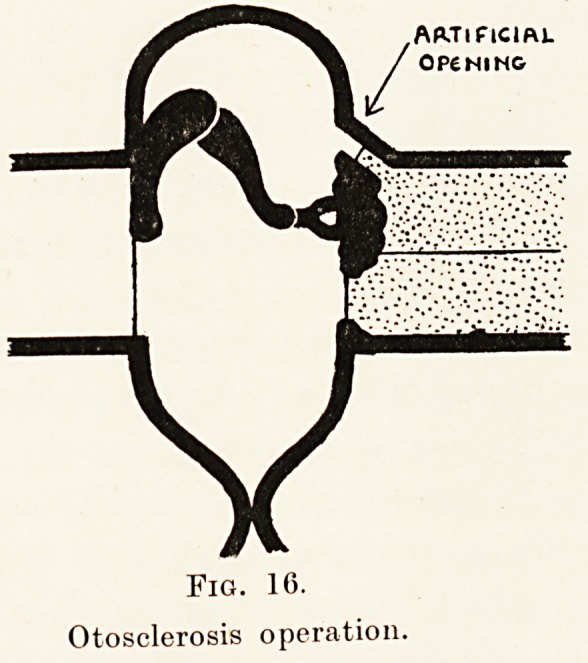# The Long Fox Lecture: The Problem of Deafness

**Published:** 1932

**Authors:** A. J. M. Wright

**Affiliations:** Lecturer in Laryngology, Rhinology and Otology in the University of Bristol


### The Bristol Medico-Chirurgical Journal

"*Scire est nescire, nisi id me*
*Scire alius sciret.*"

WINTER, 1932.

### THE LONG FOX MEMORIAL LECTURE:

DELIVERED IN THE UNIVERSITY OF BRISTOL ON OCTOBER 25TH, 1932.

THE VICE-CHANCELLOR (Dr. T. LOVEDAY, M.A., LL.D.) *in the Chair.*

BY

A. J. M. WRIGHT, M.B., B.S. Lond.,
M.B., CH.B. Brist., F.R.C.S.,

*Lecturer in Laryngology, Rhinology and Otology in the University of Bristol,*

ON

### THE PROBLEM OF DEAFNESS.

I FEEL deeply the honour which has been conferred upon me in inviting me to give this lecture. The late Dr. Long Fox would, however, I am sure, have welcomed the appointment of a laryngologist, since both his son-in-law and grandson have made such valuable contributions to this branch of medical science. The subject selected is one which has a very wide appeal. Deafness is a terrible affliction, and one which leads to more human misery than even

JAN 14 [illegible]

blindness. The old saying that there are only two classes of deafness, those which can be cured with the syringe and those which cannot be cured at all, embodies a reproach to the otologist which would on superficial examination seem to be justified. My intention this afternoon is to try to demonstrate that some advance has been made, and that there is hope for further progress in the future.

Before considering the problem of the deaf we must briefly consider the normal mechanism of hearing. The faculty of hearing is the most recent addition to the special senses, and I believe it is this fact that renders the organ of hearing particularly liable to degeneration and disease. We all, I think, appreciate this, although perhaps sub-consciously, in that we expect the aged to develop a nerve deafness, while we do not, however, expect them to develop a degeneration of the nervous mechanism of vision. Also, as in some families greyness of the hair occurs at an early age, so in others does, I believe, a premature ageing of the auditory apparatus take place. We also all recognize the sensitiveness of the auditory nerve mechanism to injury, in that we expect a dose of quinine, an overdose of alcohol or a loud sound to give us a sensation of buzzing in the ears. It will, I think, render the appreciation of how we hear more easy if we briefly review the development of hearing as we see it presented in the lower orders.*

The lowest form of life in which the faculty of hearing, though of a very low order, has been recently identified is in some species of fish. (Fig. 1.) In them the primitive sense of hearing is centred in the otolith

* I am indebted for the detail of the evolution of the ear and for Figs. 2—5 to R. T. Beatty, *Discussion on Audition*—Physical Society, 1931.

organs, the primary function of which is the indication of position, with hearing added at a later stage as a secondary function. These otolith organs consist essentially of a grouping of nerve cells with hair-like processes, on which are placed granules of calcium carbonate. These hair cells are connected by nerves with the central nervous system. During changes of position variations in pressure effects are produced on the hair cells and by them conveyed to the brain. As far as hearing is concerned the organ is a primitive one, the hair cells being directly stimulated by the sound vibrations, but obviously such an apparatus does not permit of any analysis of sound, *i.e.* there is no tone perception.

As we ascend the scale we find in amphibians (Fig. 2) the development of new groups of sensory cells designed to serve the auditory function alone, that is, we have the development of an internal ear or cochlea. In addition, we find the appearance of a tympanic membrane let into the surface of the skull and connected by a bony shaft, the columella, to a membraneous window in the wall of the cochlea. Thus, we have both an apparatus for receiving and for perceiving the sound waves. In birds and reptiles (Fig. 3) we find for the first time an arrangement in the cochlea designed for the differentiation of pitch, similar in nature to that met with in man. This consists in an orderly arrangement of nerve cells placed upon a vibrating membrane instead of upon the bone. It is of interest to note that in one particular family, the snakes (Fig. 4), whose body is not supported by limbs but is in close contact with the ground, instead of the middle ear arrangement of membrane and columella we find the columella attached directly to one of the bones of the skull. This arrangement

Fig. 1.
Fish.

Fig. 2.
Amphibian.

Fig. 3.
Bird.

Fig. 4.
Snake.

Fig. 5.
Man.

is almost certainly provided to enable them to perceive earth-borne rather than air-borne vibrations, *i.e.* a snake will perceive a footfall by vibrations conducted through the earth to the bones of its head. Two other interesting examples of adaptation of the auditory apparatus to environment are found in amphibious mammals, such as the seal, and aquatic mammals such as the whale. In both cases it is necessary to provide protection for the ear-drum during immersion. In the seal this is provided automatically by the presence of a valvular meatus which is closed by the pressure of the water. In the whale, in which, owing to its deep diving habits, pressures of as much as half a ton per square inch have to be provided against, the meatus is closed entirely, the ossicles being fixed to the bones of the skull. In this case, presumably, bone conduction is the only path of entry.

In man and other mammals (Fig. 5) we find the columella replaced by a chain of three ossicles, the malleus, incus and stapes, with a further development in length of the cochlea, which becomes wound into a spiral, presumably to allow of neatness in packing.

Following this brief account of the evolution of the organ, we must now consider in rather more detail the structure and method of working of the apparatus in man. Anatomically, we have the pinna or external ear, which has little if any function, although in many of the lower animals the concavity and mobility of the external ear is of the greatest importance in collecting and resonating the sound waves and in giving a sense of their direction. The meatus is long and sinuous, to allow of the delicate drum and nerve apparatus being placed deeply in the skull out of harm's way. The drum or middle ear (Fig. 6) is normally a closed cavity with a valvular opening (the

Eustachian tube) to the external air, by means of which the pressure within it can be regulated. It is closed externally by a very thin tympanic membrane, and stretching from this to the cochlea or internal ear is a bridge consisting of three little bones, the ossicles (*i.e.* the malleus, incus and stapes), loosely jointed together. The movements of the membrane and ossicles can be, to some extent at any rate, controlled by two muscles, the tensor tympani and stapedius, the former of which, when in action, tightens the tympanic membrane, while the latter, by tilting the stapes, probably limits the range of its movements. The cochlea consists essentially of a tube filled with liquid, the endolymph, and contained in a bony capsule. The cochlea has stretched across it the basilar membrane, which consists of a closely-arranged layer of fine fibres which vary regularly in length and probably in tension from one end to the other. (Fig. 8.) Seated on this membrane are the sensory cells or organ of Corti. The essential parts of this organ are nerve cells which bear upon their surface hair-like processes embedded in a gelatinous membrane. The cochlea is in relationship with the middle ear by two windows, one of which, the oval window, has inserted into it the footplate of the stapes, while the other, the round window, is closed by membrane. The hair cells of the cochlea are connected with the centres of hearing in the brain. There is still some degree of uncertainty as to how this auditory mechanism functions, but I will endeavour to indicate what, in view of the results of both experimental and clinical research, is the most probable method. Sound waves enter by the air of the meatus and impinge upon the tympanic membrane. From this there are three possible paths for the

FIG. 6.
Normal middle ear.

FIG. 7.
Paths of conduction.

FIG. 8.
Basilar membrane.

FIG. 9.
Pressure wave in cochlea.

vibrations to take to reach the cochlea. These paths (Fig. 7) are :—

1. By bone conduction, the vibrations being transmitted via the surrounding bone.

2. The ossicular route, the movements of the membrane being transmitted by the ossicles to the stapes and hence to the cochlea.

3. By the aerial route, the vibrations being transmitted from the membrane across the air of the middle ear to the membrane of the round window and hence to the cochlea.

It is of importance to decide which of these routes is the more important. Route 1 by bone conduction probably plays a very minor part in hearing in man, although in some of the lower animals, as has already been said, it may be the essential path. Route 2, the ossicular path, is at first sight the obvious one, and has been regarded as the essential route until recently. It now seems probable, however, that the third, or aerial, route of conduction is of as great, if not of greater, importance.

I might, perhaps, indicate some of the evidence which supports this view. Firstly, it has long been realized that excellent, if not perfect, hearing can exist when membrane and ossicles are lost. Obstruction of the round window by pathology or experiment, however, tends to produce a high degree of deafness. The physical arrangement of the middle-ear cavity also favours the conduction of sound vibrations to the round window rather than to the stapes. If we conclude, therefore, that the elaborate ossicular machinery of the middle ear is not solely designed for the transmission of sound waves, what is its alternative function ? We have an air-containing cavity, the pressure within which is under control by the Eustachian tube. There is a bridge across this cavity formed by the ossicles and stretching from the membrane to the oval window in the wall of the cochlea. The tension of this bridge can be varied by the action of the tympanic muscles and also by the air pressure within the middle ear. Variations in this tension must produce variations in the physical conditions within the middle ear and cochlea, and it seems probable that one object of this apparatus is to provide a mechanism of accommodation by which the optimum tension can be maintained within middle ear and cochlea, and by which an adequate, but not excessive, amount of sound wave or stimulus can be passed on to the delicate nerve endings.

To turn now to the mode of action of the cochlea. Since Helmholtz propounded the idea that the perception of pitch was carried out by sympathetic vibration of particular portions of the basilar membrane (Fig. 8) to the particular tone, and thereby stimulating the hair cells lying thereon, a fierce battle has raged as to the correctness or otherwise of this view. I myself think that there is now little doubt that some such method of action occurs. There is a great deal of evidence in favour of this theory, and I propose briefly to indicate a portion of it.

It has been found that in cases of so-called occupation-deafness, in which a defect in hearing results from continued subjection to loud sounds, an area of degeneration is found in the organ of Corti, and such degeneration is constant in position for sounds of any given pitch. Similarly, it has been experimentally shown in animals that damage to the basilar membrane at any particular level will produce a defect in hearing for tones of a certain pitch and not for others. From the clinical side cases are also occasionally seen in which one is justified in diagnosing a disease in the cochlea and in which there is found a loss of hearing for particular tones. I have recently seen the most perfect example of this, nature's experiment, in support of the theory of resonance in the cochlea. The patient, during the course of an attack of mumps, became deaf in one ear. It is well recognized that occasionally mumps will produce damage in the cochlea, but such damage is, as a rule, so widespread as to destroy the hearing in the affected ear entirely. In this particular case, however, the injury to the cochlea had resulted in an absolute loss of hearing for high tones only, there being a sharp line of demarcation between those tones which were heard and those which were not. It is difficult to explain this observation on any other ground than that particular regions of the cochlea serve for hearing particular tones. We must, therefore, conceive of the cochlea as consisting of a series of strings, each one of which vibrates in response to its particular tone and transmits the sensation for this tone to the centres in the brain. These strings are immersed in fluid and contained in a cavity in dense bone. Since fluid is incompressible, a window is provided at either end of the tube to allow for passage of the sound waves, so that when the stapes is pushed inwards in the oval window the membrane in the round window can be forced outwards. (Fig. 9.)

Having thus briefly considered the normal mechanism of hearing, we must now consider what changes may take place in the production of deafness. As far as the meatus is concerned, any blockage will, of course, produce some degree of deafness, and I have nothing further to say as to this.

FIG. 10.

Acute inflammation of middle ear.

FIG. 11.
Results of inflammation (1).

FIG. 12.

Results of inflammation (2).

FIG. 13.
Otosclerosis.

### THE MIDDLE EAR.

Attacks of inflammation in the middle ear are extremely common, and are responsible for a considerable proportion of the total cases of deafness. Such inflammation, for practical purposes, always spreads to the middle ear by the Eustachian tube, its only communication with the exterior. This being the case, such conditions are always secondary to inflammations in the nose and throat. Inflammation in the middle ear may produce a defect in hearing in various ways. Thus, there may be in the acuter stages (Fig. 10) an accumulation of fluid and a swelling of the lining, while as a later result the membrane and ossicles may be destroyed (Fig. 11) in whole or in part or may be altered in structure so as to be unduly rigid or flaccid. (Fig. 12.) The windows into the cochlea may also be obstructed. Finally, as a result of obstruction to the Eustachian tube, you may have induced a positive or negative pressure within the middle ear. It will be realized, therefore, that the actual mechanical conditions in the middle ear producing a defect in hearing may be extraordinarily diverse, and we are as yet uncertain as to what mechanical conditions will produce the maximum degree of defect. Clinical experience and experimental evidence does, however, establish that interference with the windows into the cochlea is probably the most important factor. (Fig. 12.)

One great difficulty in considering the results produced by changes in the middle ear is that it is not by any means easy to decide how far the defect in hearing produced is due to interference with conduction of the sound waves through the middle ear and how far it is due to secondary changes in the cochlea. In addition to inflammation and the results produced by it in the middle ear, we have also to consider a mysterious complaint, otosclerosis. In this condition changes take place in the bone surrounding the windows, and as a result of these changes the stapes tends to become firmly fixed in its window, resulting in a high degree of deafness. This complaint, otosclerosis (Fig. 13), is responsible for a very large number of cases of deafness. It is chiefly met with in women, shows a strong hereditary tendency, and there is some evidence suggesting that some abnormality of internal glandular secretion, possibly of the parathyroids, is a factor in its causation.

### THE COCHLEA.

As has already been indicated, the organ of Corti, the essential sensory organ in the cochlea, is extremely easily damaged by disease or injury. Just as in the eye the maintenance of correct pressure of the fluids contained in it is essential for the proper functioning of the nerve endings (any undue increase of pressure resulting in the condition known as glaucoma), so in the cochlea is this factor probably of even more importance. Owing to the fact that the cochlea is embedded in dense bone within the skull, it has, up to the present, not been possible to obtain any experimental evidence as to variations of the pressure of its contained fluid. I believe, however, that such variations in pressure are of the utmost importance in the pathology of deafness. Some variation in pressure probably results from a vast variety of causes. As has already been indicated, changes in pressure in the middle ear are certainly to some extent transmitted to the cochlea. To give a simple example of how easily pressure changes are induced in the cochlea, I can mention the familiar fact that hanging the head will, in most individuals, induce a buzzing in the ears, the explanation probably being simply an attempt to overfill the cochlea by the distended blood vessels. This being the case, it will be understood that any complaint which affects the general circulation or the general balance of tissue fluids may produce changes in the pressure within the cochlea and therefore a defect in hearing. It seems probable that changes in pressure, if of short duration, will produce merely a transient defect in hearing. On the other hand, if long continued, it is probably followed by a degeneration of the nerve elements and therefore by a permanent defect.

I have observed in a long clinical experience of some cases of deafness that they tend to exhibit periods in which the hearing improves and periods in which it becomes worse, without any accompanying change in the middle ear to account for this, and I believe that such cases are to be explained by intermittent variations in pressure.

Apart from pressure changes in the cochlea, cases of actual damage to the organ by disease or injury are, of course, not infrequent. As an example of injury, any unusually loud sound, such as an explosion or prolonged sound above a given intensity, tends to produce degeneration of the organ. As an example of disease, one can instance syphilis, in which both the congenital and acquired varieties may produce widespread destruction of the nerve elements.

What can be done to prevent or remove these various pathological conditions ? As far as the meatus is concerned, the problem is simply one of maintaining an open tube. In the case of the middle ear we have to consider the essentially different problems of the prevention or control of inflammatory changes and otosclerosis. Inflammatory changes are amenable in practically all cases during the early stages, and the key to the prevention of such cases of deafness in the adult lies in the early and efficient treatment of the primary inflammatory condition in childhood. Much has been done in this direction, and no doubt more will be done in the future. The medical inspection of school children, with the resulting early treatment of those cases in which deafness is apparent, is producing fruitful results, as is also the systematic treatment of ear complications in cases of acute specific fevers, such as scarlet fever and measles, in isolation hospitals. During the last few years the public authorities have, in the larger centres, appointed specialists on diseases of the ear to deal with such cases, and, as a result, one is seeing fewer and fewer cases in which a suppuration in the ear resulting from scarlet fever has been allowed to drift on into a chronic condition with resultant damage to the hearing.

In Glasgow Dr. Ker Love[1] has had an opportunity of reviewing the percentage of acquired cases of deafness in school children during the last forty years, and he finds that such percentage has dropped by one half. In addition to more efficient treatment in the early stages, he rightly attributes this improvement to improved social conditions, *i.e.* better housing, feeding and clothing, etc. Some five years ago I analysed my private records, covering a period of sixteen years, to try and ascertain to what degree we had succeeded in the prevention of chronic ear disease. These figures seemed definitely to show that the incidence of chronic suppurative disease in the middle ear is decidedly on the down grade. They applied to private cases in which the factors of school inspection and improved social conditions did not operate, so that in them we must give the credit to more systematic treatment.

Apart from the ideal of prevention, what can be done towards alleviating the deafness resulting from old-standing inflammation? While something has been done, it is here I feel that there is room for considerably further clinical research. Until further light has been thrown on the exact method by which the machine works—and this I feel is most likely to result from careful clinical research into a large number of cases—our methods of treatment in any individual case are necessarily experimental. That there is hope in the future that much may be done is, I think, suggested by the following isolated observations:—

1. In some cases of middle-ear deafness, if an opening is made artificially in the membrane a dramatic improvement in hearing ensues.

2. In some cases in which there is already a hole in the membrane, if this be closed temporarily by a patch or a plug, forming the so-called artificial drum, the hearing is again sometimes dramatically improved. It has seemed to me that the startling results sometimes obtained by the use of an artificial drum should, if correctly interpreted, throw a considerable amount of light on the mechanism of hearing. I have tried to carry out some research into a few such cases, and perhaps it might be of interest briefly to detail the results of two typical experiments. In the first of these (Fig. 14) the closure of a small hole in the tympanic membrane by means of a piece of cigarette paper produced a very considerable improvement in the hearing. I suggest that in this case the improvement is due to the re-conversion of the middle ear into a

FIG. 14.
Artificial drum (1).

FIG. 15.
Artificial drum (2).

FIG. 16.
Otosclerosis operation.

closed air-containing cavity, thereby enabling the normal conduction of sound waves by the aerial route across the middle ear to be re-established. In the second experiment (Fig. 15), in which there was an extensive loss of tympanic membrane, the introduction of fluid into the middle ear cavity to such a level as would cover the round window produced improvement in the hearing. Further filling up to the level of the stapes in the oval window produced a lowering of the hearing. I suggest that this observation can be explained as follows. Before the introduction of the fluid sound waves entered the cochlea by the round and oval windows simultaneously. These waves would tend to meet and neutralize (see Fig. 9), thereby diminishing the effect on the nerve endings. The screening of one window, however, by the liquid prevents this.

Treatment directed towards restoring the patency of the Eustachian tube or, as far as possible, towards the replacement of the membrane and ossicles in their correct position, has occasionally produced good results; but, as a whole, it seems to me that progress is likely to lie in the direction of getting rid of stiffened and ill-acting structures in the middle ear, rather than in attempting the usually impossible task of restoring them to efficiency.

As far as our knowledge goes at present, what seems to decide the possibility of improvement or not, as far as the middle ear is concerned, is the efficiency of the structures in the windows into the cochlea rather than the condition of the structures in the middle ear itself. This fact brings us on to the consideration of otosclerosis, which, while a problem in itself, does resemble the mechanical conditions presented in many cases of inflammatory disease, in that it results in the blocking of one of the windows.

### OTOSCLEROSIS.

As far as otosclerosis is concerned, two problems await solution. Firstly, the exact nature and mode of origin of the disease must be ascertained, and it is then possible that treatment may do something towards preventing its development in the individual case. Until this has been ascertained all that can be done in the way of prevention is by the limitation of reproduction in families in which the disease occurs. Secondly, efforts must be directed towards removing the mechanical results of the disease, and thereby improving the hearing. Some degree of success has already been made at the hands of Sourdille[2] by the provision of a new artificial opening in the wall of the cochlea. (Fig. 16.) This idea is not a new one, but it will be understood that the technical difficulties involved in making a new window and in maintaining its patency are very great.

### WHAT HAS BEEN DONE AND WHAT MAY BE DONE IN THE FUTURE IN REGARD TO THE COCHLEA?

It should be possible to prevent damage by loud sounds by taking precautions to prevent their access to the cochlea. As an example of what I mean I may instance the closure of the meatus by special appliances during gun firing. We all know that attempts have been made to delay the onset of senility, and should such attempts prove successful we shall no doubt have no longer to shout at the aged! It has been said that many diseases may affect the nervous structure of the cochlea, and syphilis was instanced as an example. A very considerable proportion of cases of extreme deafness in children and in adults is due to syphilis, and on the whole treatment does little in such cases, at any rate when once signs of damage to the ear become apparent. Prevention of this type of deafness must, therefore, lie in the direction of further efforts towards the total abolition of the disease.

The problem of treatment in cases in which defects in labyrinthine tension are an important factor is similar to that involved in general constitutional defects such as arteriosclerosis, which seem to result from the very unphysiological lives lived by so many of us, in which over-eating, over-smoking, and excesses of all kinds play such a large part. Prevention here lies in the direction of a more simple and natural life, but the treatment of such conditions when established is difficult. The correction of errors in living is often only possible to a limited degree, and is productive of only a limited degree of improvement. Some particular drugs, however, of which pilocarpine is an example, do seem to have some direct action on the labyrinthine pressure, and it seems possible that further progress may be made in this direction.

As a last resort, in cases in which the hearing is so defective that conversation cannot be carried on or only with great difficulty, mechanical aids may be of the greatest utility. The deaf have always been the natural prey of charlatans, and the advertising columns of any cheap newspaper will demonstrate that this is still the case. The principles underlying aids to hearing are simple. Such aids can be classified into two classes: electrical and non-electrical. Dealing first with the second group, non-electrical aids, these are represented in the simplest form by the conversation tube, which consists of a tube conducting the sound waves direct to the ear. In other varieties the use of an expanded end as a sound collector to receive the waves increases their efficiency. In yet others this expanded end, by being made flask-shaped, introduces an element of magnification by resonance. Broadly, the larger the non-electrical aid the more efficient it is likely to be, and any small non-electrical instrument which can be introduced into the meatus must be useless.

*Electrical Aids.*—Until recently these all depended on the conversion of sound waves into electrical ones with their magnification and reconversion to sound waves, *i.e.* the micro-telephone. With this machine it is possible to obtain extreme degrees of magnification, but there is usually some distortion introduced and some tones are magnified more than others. This defect does, however, introduce the possibility of further improvement. In the majority of cases of deafness the defect in hearing is most marked for particular tones, and the time may not be far distant when it will be possible to fit any given patient with an electrical apparatus which will magnify those tones which are least well heard.

Another entirely different principle has recently been employed by which the sound waves are magnified by a wireless amplifying circuit. This arrangement produces the maximum of magnification with the minimum of distortion, but unfortunately the apparatus is so cumbersome as to prevent its being used except in one location.

In conclusion, a word as to the mental side of deafness. It requires a mental effort to use any sensory organ which is defective, and many deaf people, owing to the effort involved, give up trying to hear. It is therefore of the utmost importance both to encourage them to listen and by aids, or any other means within one's power, to persuade them to exercise their hearing to the greatest possible degree. By this alone it is possible in many cases not only to prevent the hearing from further deteriorating but in some degree to improve it.

REFERENCES.

[1] *Jour. Laryng. and Otol.*, 1932, xlvii. 556.

[2] *Ibid.*, 1930, xlv. 601.

## Figures and Tables

**Fig. 1. f1:**
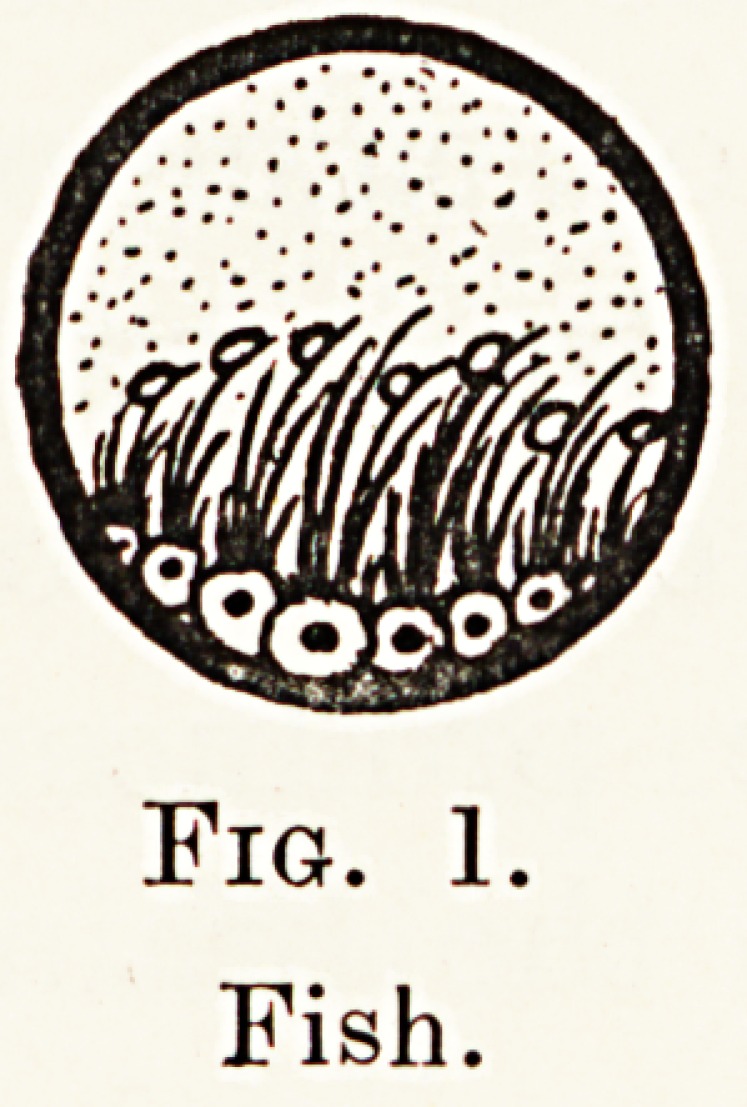


**Fig. 2. f2:**
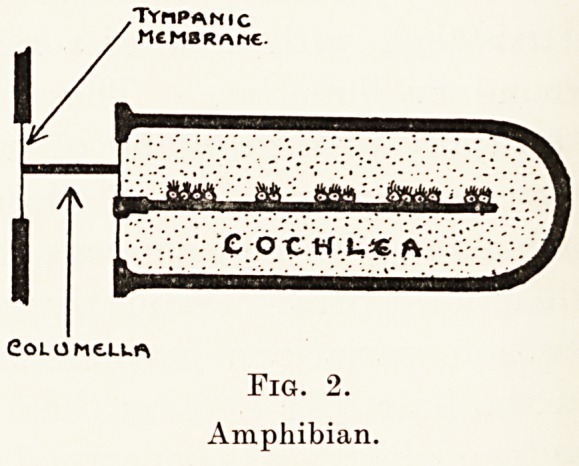


**Fig. 3. f3:**
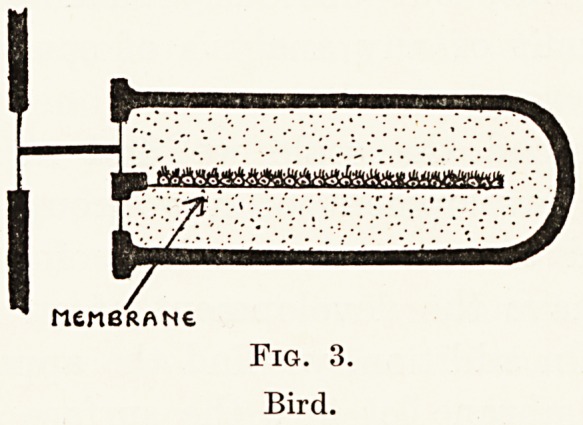


**Fig. 4. f4:**
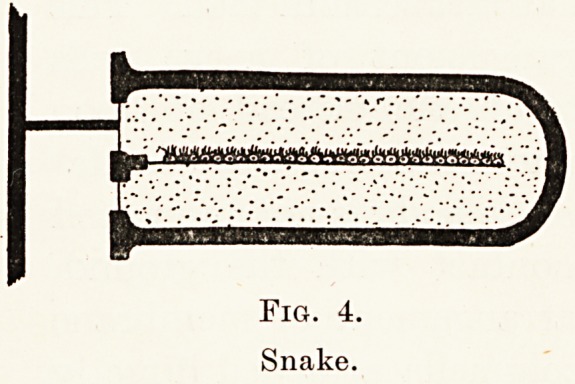


**Fig. 5. f5:**
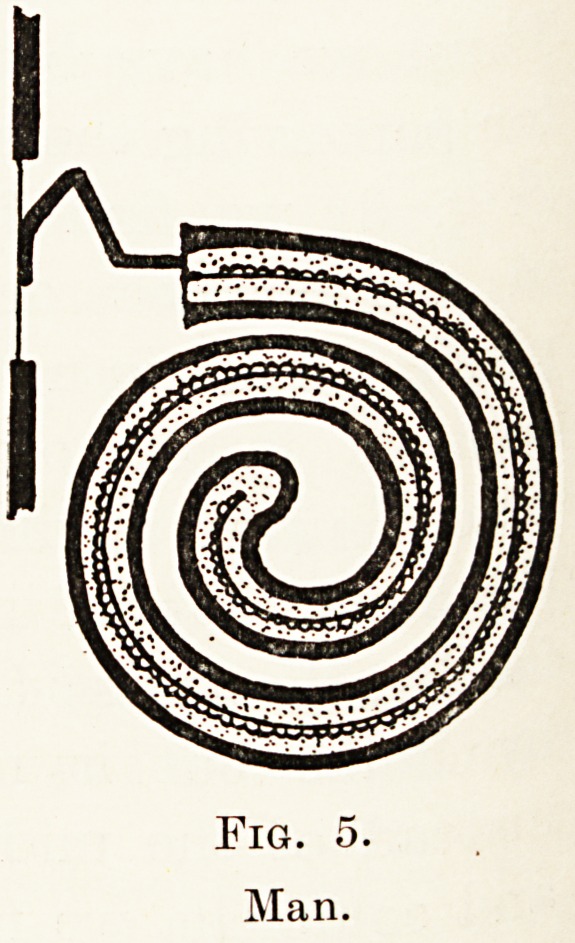


**Fig. 6. f6:**
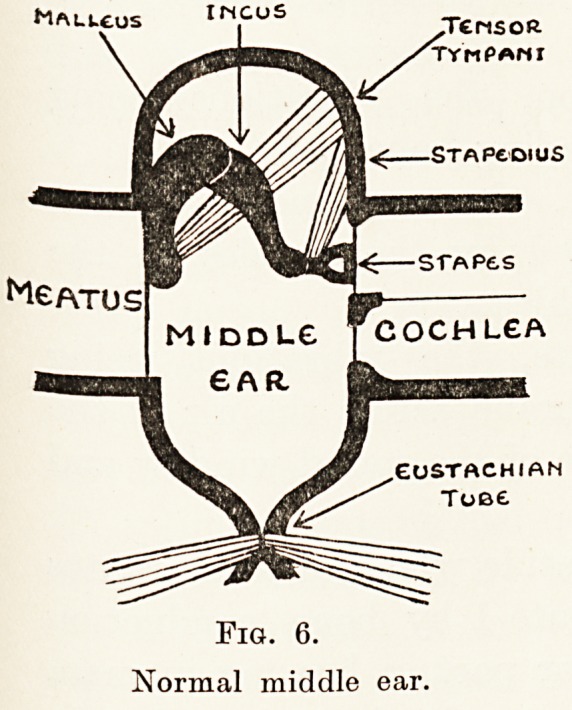


**Fig. 7. f7:**
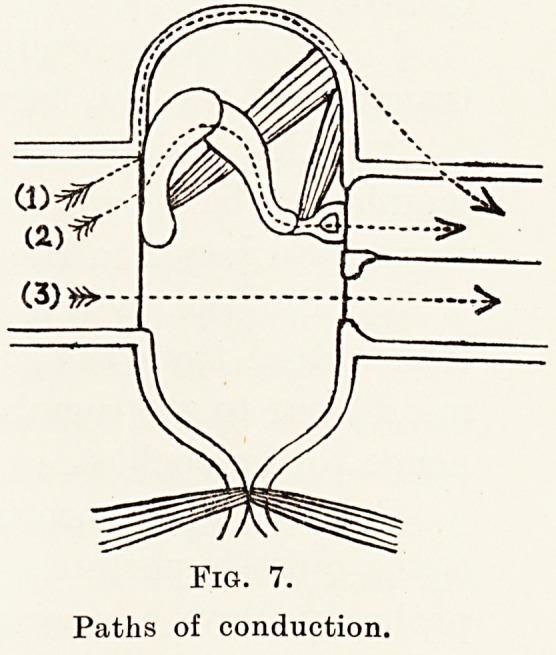


**Fig. 8. f8:**
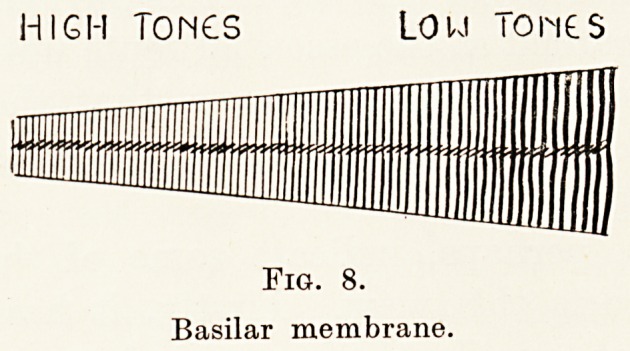


**Fig. 9. f9:**
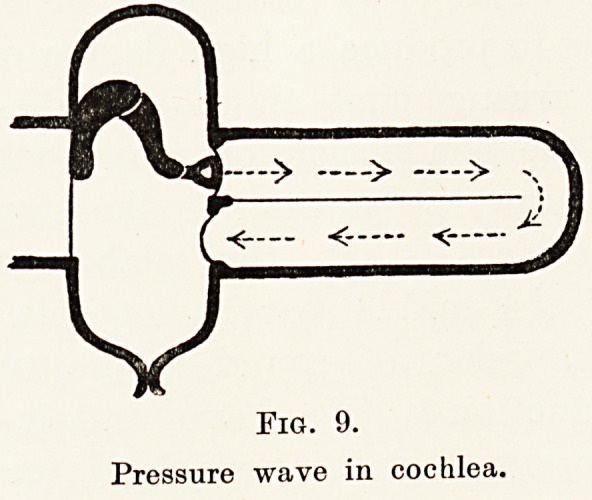


**Fig. 10. f10:**
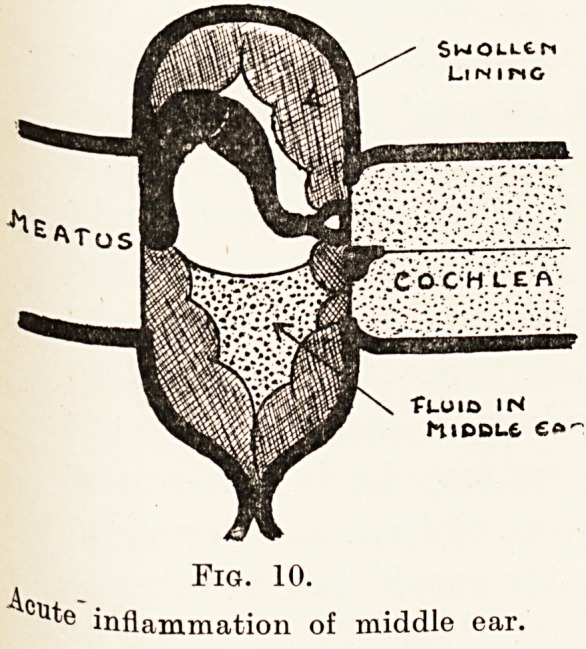


**Fig. 11. f11:**
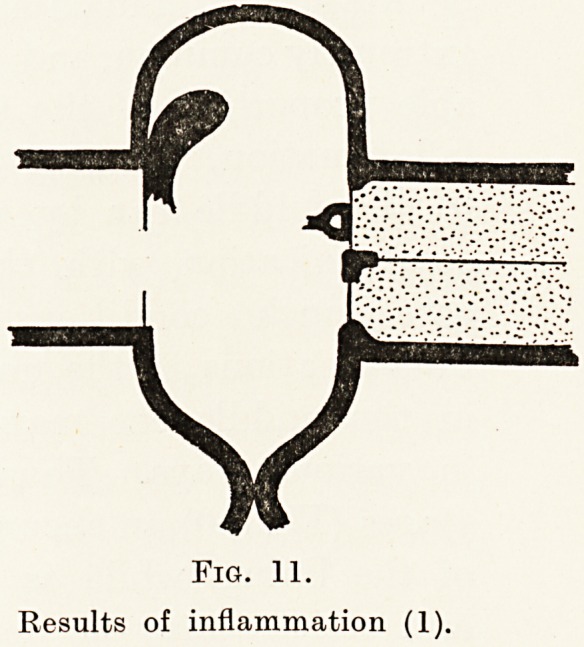


**Fig. 12. f12:**
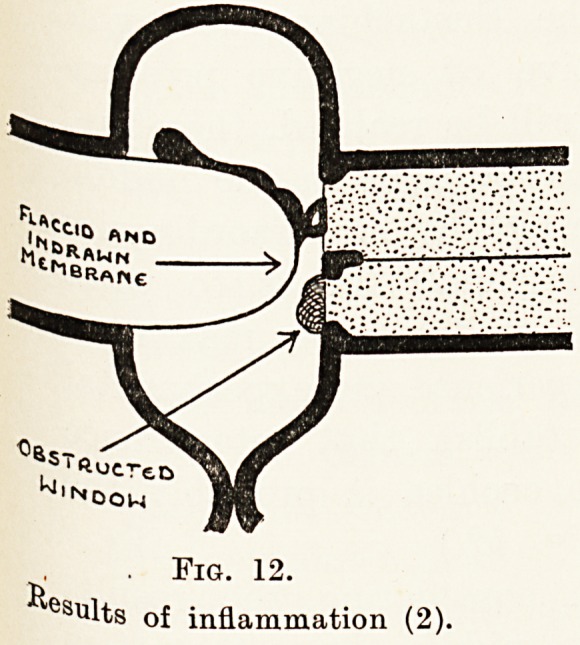


**Fig. 13. f13:**
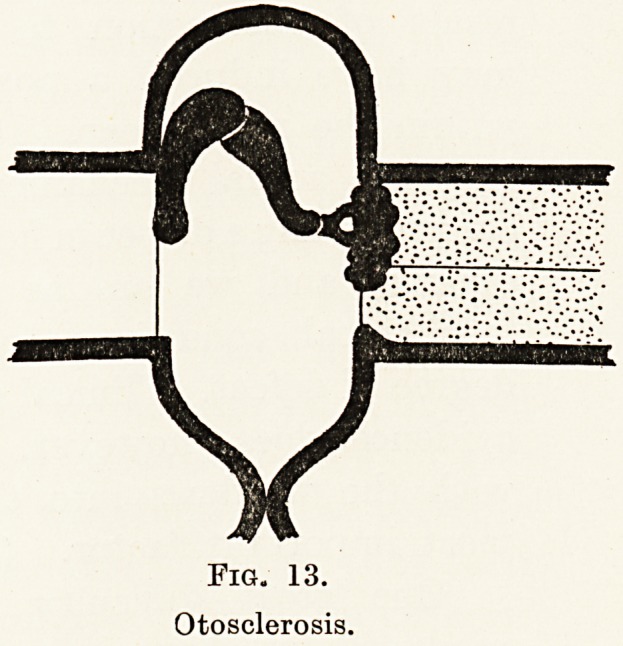


**Fig. 14. f14:**
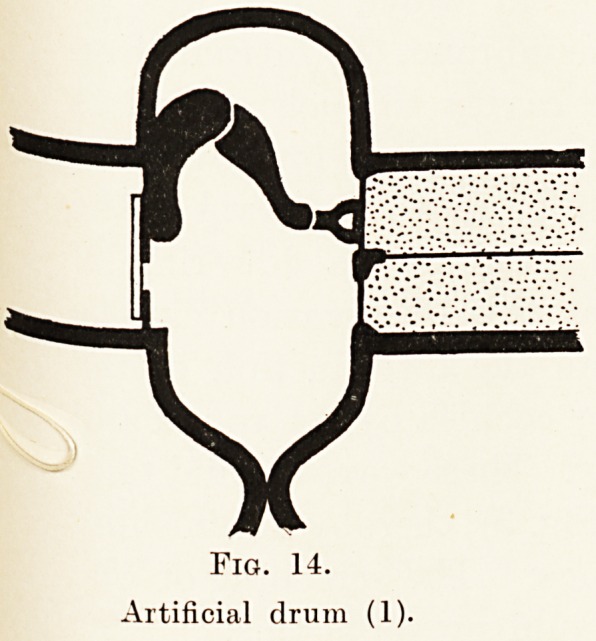


**Fig. 15. f15:**
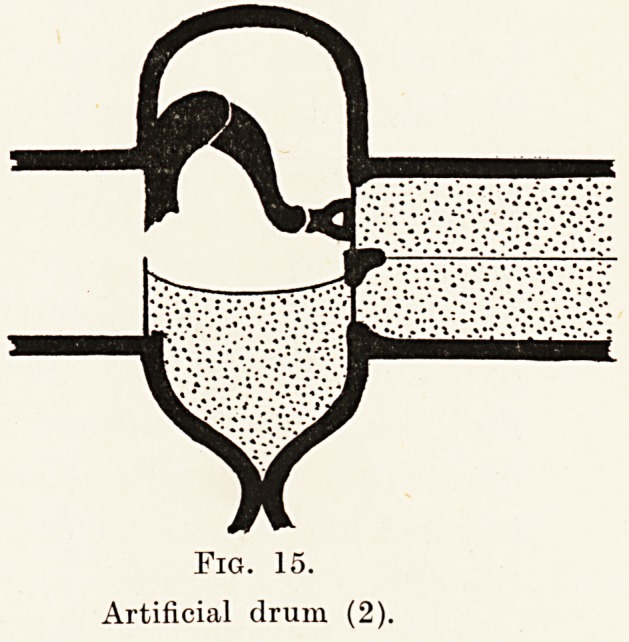


**Fig. 16. f16:**